# Head lice as vectors of pathogenic microorganisms

**DOI:** 10.1186/s41182-023-00545-5

**Published:** 2023-09-20

**Authors:** Hermann Feldmeier

**Affiliations:** Institute of Microbiology, Infectious Diseases and Immunology, Charité-Universitätsmedizin Berlin, corporate member of Freie Universität Berlin, Humboldt-Universität zu Berlin, and Berlin Institute of Health, Campus Benjamin Franklin, Hindenburgdamm 30, 12203 Berlin, Germany

**Keywords:** *Pediculus humanus*, Head lice, Body lice, Bacterial pathogen

## Abstract

Body lice and head lice are the most common ectoparasites of humans. Head lice (*Pediculus humanus capitis*) occur worldwide in children and their caretakers, irrespective of their social status. In contrast, body lice (*Pediculus humanus corporis*) are confined to marginalized population groups in countries of the Global South, homeless people, and refugees. Body lice are known to transmit an array of bacterial pathogens, such as *R. prowazekii*, *R. rickettsii*, *C. burneti*, *B. quintana*, *B. recurrentis*, and *Y. pestis*. The vector capacity of head lice is still a matter of debate. The objective of the review was to scrutinize the existing evidence on the vector capacity of head lice for the transmission of bacterial pathogens. The PUBMED database was searched using a combination of the terms “pediculus humanus” OR “body lice” OR “head lice” AND “pathogen” OR “Rickettsia prowazekii” OR “Bartonella quintana” OR “Borrelia recurrentis” OR “Coxiella burneti” without a time limit. Data from epidemiological studies as well as historical observations demonstrate that body lice and head lice can carry the same array of pathogens. Since the presence of a bacterial pathogen in an arthropod is not sufficient to state that it can be transmitted to humans, and since experimental models are lacking, as yet one cannot conclude with certainty that head lice serve as vectors, although this review presents circumstantial evidence that they do. Adequately designed experimental and epidemiological studies are needed to ascertain the exact transmission potential of head lice.

## Background

Human lice, *Pediculus humanus,* have been known as human ectoparasites for thousands of years. They have been identified at burying sites in Israel from 60 A.D. [1] and in mummies from Amerindians buried in Peru at an altitude of 3000 m around 3000 years ago [2]. Lice are obligate blood-sucking parasites. Phylogenetically, they belong to different mitochondrial clades of which the geographical distribution shows some differences, although overlapping of clades is common [3, 4]. Head lice occur globally and affect millions of children and their caretakers, irrespective of their social level. In contrast, nowadays body lice infest only marginalized population groups, such as homeless people and refugees, and occur in special settings such as in impoverished communities situated at high altitude in Ethiopia or in prisons in low-income countries, where hygienic conditions are poor, and crowding is common [5]. Until a few years ago, scientists assumed that only body lice can transmit bacterial pathogens. If head lice have a similar vector capacity for the transmission of important bacterial pathogens, then the potential health threat caused by head lice infestation should be several orders of magnitude greater compared to body lice. The objective of the review was to summarize the existing knowledge on the vector capacity of head lice for the transmission of bacterial pathogens in a comprehensive manner.

## Materials and methods

### Search strategies and selection criteria

**Fig. 1:**
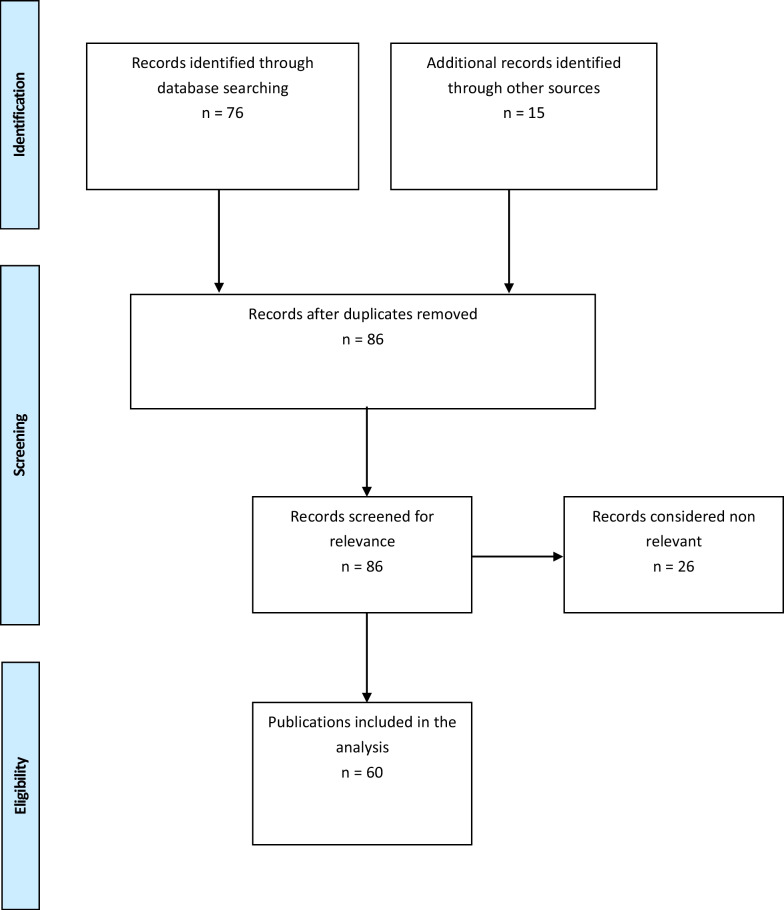
Flow diagram of the literature search

The results of this study are reported according to the Preferred Reporting Items for Systematic Reviews (PRISMA). The PUBMED database was searched using a combination of the terms “pediculus humanus” OR “body lice” OR “head lice” AND “pathogen” OR “Rickettsia prowazekii” OR “Bartonella quintana” OR “Borrelia recurrentis” OR “Coxiella burneti” without a time limit. Additional searches were undertaken in textbooks. Reference lists of papers included in the analysis were manually searched. Historical or anecdotal articles were taken from the library of the author. The abstracts of the articles were screened for relevance with regard to the objective of the study. The full-text articles were reviewed for eligibility. Articles were considered eligible when methods and results were described comprehensively and adequately (see flow chart Fig. 1).

### Data extraction and analysis

Data extracted included study origin, design of the study, characteristics of settings and participants, and results. All data extracted are summarized in Tables 1, 2 and 3 and narratively described in the text.

**Table 1:** Experimental studies confirming body lice as vectors of bacterial pathogens

Pathogen	References
*R. prowazekii*	[20, 49, 52]
*R. rickettsii*	[53]
*R. conorii/R. typhi*	[54]
*B. quintana*	[49, 55]
*B. recurrentis*	[49, 56, 57]
*Yersinia pestis*	[7, 58]
*Acinetobacter spp.*	[59, 60]

**Table 2:** Epidemiological studies in population groups infested with head lice

Country; area/city	Year	Study design/detection of DNA of	Number of persons/lice examined	Number of lice positive (%)	Pathogens identified	Remarks	References
Democratic Republic of Congo; Kinshasa	2019	Examination of patients hospitalized at the Monkole Hospital Center/*B. quintana, B. recurrentis, R. prowazekii, Anaplasma spp., Y. pestis, C. burneti, Acinetobacter spp.*	27/181	54/181 (29.8%)	*A. baumannii; A. johnsonii; A. sai; A. pittii; A. guillouiae; A. pediculi*	Head lice belonged to clade A, D, E; 44% of the infested patients had head lice from different clades	[28]
Madagascar; rural communities in the Southeast	2019	Collection of head lice from inhabitants of rural communities/*B. quintana, Acinetobacter spp.*	33/151	20/151 (30.2%)	*B. quintana* 12.6%; *Acinetobacter* 42.1%	Frequency of pathogens varied considerably between villages	[23]
Algeria; Algier	2019	Collection of head lice from refugees from Niger and school children/ *Rickettsia spp.,* *Borrelia spp.,* *B. quintana;* *Y. pestis,* *C. burneti;* *Anaplasma spp.;* *Acinetobacter spp.*	70 refugees, 101 school children/ 37 lice from refugees, 45 lice from school children	*C. burneti*: lice positive school children 0/45 (0%)] refugees 3/31 (9.7%) Acinetobacter spp. school children 25/45 (55.6%) refugees 25/31 (80.6%)	*C. burneti;* *A. baumanii;* *A. johnsonii;* *A. variabilis*	Head lice belonged to clade A, B, E	[25]
Mali; Koulikoro region	2017	Collection of head lice from patients presenting at rural health centers Patients were examined for presence of head and body lice; only head lice were present/*B. quintana, Rickettsia spp., Anaplasma spp.*	117/600	*B. quintana* lice positive 3/600 (0.5%) *C. burneti*: lice positive 6/117 (5.1%) *Rickettsia spp.* lice positive 4/600 (0.6%)	*B. quintana* *C. burneti* *Rickettsia spp.* *Anaplasma spp*.	Lice belonged to clade E but showed many different haplotypes	[24]
Democratic Republic of Congo; tropical rain forest inhabited by pygmies	2016	Collection of head lice from healthy individuals of 3 communities/ *Borrelia spp.,* *Bartonella spp.,* *Acinetobacter spp.,* *Rickettsia spp.,* *R. prowazekii,* *Y. pestis,* *Anaplasma spp*.	120/630	lice positive 246/630 (39.0%) Borrelia spp. 11/630 (1.7%) (*B. recurrentis* 10/11 (90.9%) *B. theileri* 1/11 (9.1%),	*Borrelia spp.* *A. junii,* *A. ursingii,* *A. baumannii,* *A. johnsonii,* *A. schindleri,* *A. lwoffii,* *A. nosocomialis,* *A. towneri,* *Moraxella spp.*	lice belonged to clade A, C, D; distribution of clades differed between villages	[26]
Thailand; schools in different areas of the country	2015	Head lice collected from school children/*Bartonella spp., Acinetobacter spp.*	26/275	lice positive 10/275 (3.7%)	*A. baumannii,* *A. radioresistens, A. schindleri*		[29]
Ethiopia	2013	Head lice collected from patients with louse-borne relapsing fever	24/35	Lice positive 8/35 (23.0%)	*B. recurrentis*		[27]
France	2011	Head lice collected from school children during an epidemiological survey/ *B. quintana* *A. baumannii*	?/288	*A. baumannii* lice positive 95/288 (33.0%)	*A. baumannii*		[30]

**Table 3:** Epidemiological studies in population groups co-infested with head lice and body lice

Country; area/city	Year	Study design/detection of DNA of	Number of persons/lice examined	Number of lice positive (%)	Pathogens identified	Remarks	References
France; town near Paris	2018	Head lice and body lice were collected from in-patients of the Avicenne Hospital and homeless people/*Acinetobacter* spp.; *R. prowazekii*; *Y. pestis*; *Borrelia* spp.; *B. quintana*; *C. burneti*; *Anaplasma* spp.	141/head lice: 235; body lice: 24	*B. quintana*: Head lice: 0/235 (0%) Body lice: 4/24 (16.7%) *Acinetobacter* spp.: Head lice 27/235 (11.5%) Body lice 7/24 (29.1%)	*B. quintana* *Acinetobacter* spp.	Lice belonged to Clade A, B, E	[3]
Democratic Republic of Congo; Oriental province	2015	Collection of head lice and body lice from healthy individuals living in a *Y. pestis* endemic area/*B. quintana*, *Y. pestis*	37/examined for *B. quintana* Head lice: 7 Body lice: 30 Examined for *Y. pestis* Head lice: 31 Body lice: 143	*B. quintana* Head lice: 6/31 (19.4%) Body lice: 48/143 (33.5%) *Y. pestis* Head lice 1/31 (3.2%) Body lice 2/148 (1.4%)	*B. quintana*, *Y. pestis*	Lice of 7 persons were infected with *B. quintana*, *Y. pestis* or both pathogens; Clade A contained head lice and body lice; Clade D contained only body lice	[4]
USA, San Francisco	2014	Collection of head lice and body lice from self-selected homeless people/*B. quintana*	203/head lice: 10/203; body lice: 60/203; both types 6/203	Head lice: 37.5% Body lice: 15.9%^a^	*B. quintana*		[50]
Ethiopia; Bahir Da Hospital	2013	Examination of head lice + body lice collected from in-patients in whom *Borrelia spp*. were identified microscopically in blood smear/*B. recurrentis*	24/35 head lice; 62 body lice	Head lice: 8/35 (22.9%) Body lice: 25/62 (40.3%)	*B. recurrentis*	Clades not determined; in co-infested patients *B. recurrentis* DNA was detected more often in body lice than in head lice	[27]
Democratic Republic of Congo; Oriental province, Rethy Health District	2013	Head and body lice were collected from individuals living in a *Y. pestis* endemic area/*R. prowazekii*, *B. recurrentis*, *B. quintana*, *Y. pestis*	Number of persons not detailed/35 head lice; 154 body lice	*B. quintana*: Head lice 6/35 (17.1%) Body lice 50/154 (32.5%) *Y. pestis* 1/35 head lice (2.9%) 2/154 body lice (1.3%)	*B. quintana* *Y. pestis*		[31]
Ethiopia; 8 villages located at different altitudes in SW Ethiopia	2011	Collection of head lice + body lice from individuals living in a *B. quintana *endemic area/*B. quintana*	134/head lice:271; body lice: 424	Head lice: 19/271 (7.0%) Body lice: 76/424 (17.9%	*B. quintana*	Head lice belonged to clade C, body lice belonged to clade A; in co-infested persons only head lice or only body lice were infected with *B. quintana*	[32]
Burundi; various sites	2002	Lice were collected during an outbreak of epidemic typhus in Burundi/*R. prowazekii*, *B. quintana*	No data provided/no data provided	Body lice: *R. prowazekii* 7–35% *B. quintana* 2–90% Head lice: no data provided	*R. prowazekii* *B. quintana*	Not clear how many body and head lice were collected/examined	[20]
Nepal; Pokhara town + slum in Katmandu	2006	Lice were collected from healthy children/*B. quintana*	No data provided/no data provided	Town: Head lice: 0% Body lice: 12.5% Slum: Head lice: 25% Body lice: 19%	*B. quintana*		[21]
USA; San Francisco	2009	Head and body lice were collected from homeless persons/*B. quintana*	138/no data provided	Head lice: 8% Body lice: 5%^b^	*B. quintana*		[22]
Ethiopia; rural communities in the Southeast	2012	Head and body lice collected from healthy individuals/*A. baumannii*	134/head lice: 115; body lice: 109	Head lice: 54/115 (47%) Body lice: 77/109 (71%)	*A. baumannii*		[34]
Algeria, Mali, Senegal, Ethiopia, Democratic Republic of Congo, Rwanda, Burundi, Kenya, Madagascar	2014	Not details provided where and how lice were collected/*B. quintana*	Not detailed/1040 lice in total; head lice: 616; body lice: 424^c^	Head lice: 10/616 (1.6%) Body lice: 228/424 (54.0%)	*B. quintana*	Proportion of head and/or body lice infected differed considerably between settings Range head lice: 0–17.1% Range body lice: 4.5–89.7% All head and body lice infected with *B. quintana* belonged to clade A2	[33]

^a^Head lice and body lice were examined as pools

^b^Positivity rate from pools of lice

^c^Absolute and relative number of head and body lice differed considerably between collection on sites; in some sites only head lice were collected

## Main text

### Historical evidence

In 2001, a mass grave of Napoleon’s soldiers was detected in Vilnius, Lithuania. About 3000 soldiers had obviously been buried in a hurry, the skeletons being in close proximity and in positions indicating that they were buried before rigor mortis had set in [6]. At a site where 717 individuals were buried in a trench, teeth were extracted from corpses and remains of five lice were recovered. Using suicide PCR in the pulpa of 4 out of 72 teeth, DNA of *R. prowazekii* was identified. The recovered lice were considered to be body lice on the basis of morphology and sequence data [6]. *B. quintana* DNA was identified in ten teeth and three lice.

The hygienic conditions during the retreat of Napoleon’s army in the winter of 1812 must have been very poor and crowding in shelters where the soldiers passed the nights must have been intense, making the propagation of both head lice and body lice very likely. Hence, if the soldiers who died from typhus obviously were infested with body lice, it is highly probable that they were also infested with head lice.

There is circumstantial evidence that in the Second Plague Pandemic in Europe (fourteenth–nineteenth century), including the period of the Black Death (1346–1353), *Pediculus humanus* acted as vector for *Yersinia pestis* and that the role of fleas in the transmission of the pathogen was overestimated for centuries [7]. Historical data convincingly indicate that during the period of the Second Plague Pandemic the great majority of people were extremely poor and lived in miserable conditions. Poor hygiene was the rule and head lice and body lice were extremely widespread [8]. Using a compartment model for plague transmission kinetics, Dean et al. [9] showed that transmission by *Pediculus humanus* fitted significantly better to the mortality curves from nine local outbreaks during the Second Plague Pandemic than models for airborne transmission (as it occurs in pneumonic plague) or rodent transmission. Even at the end of the nineteenth century, in cities, such as Hamburg, Germany, *Pediculus humanus capitis* was rampant in impoverished population groups, and head lice occurred in all age groups [10].

In World War I, a relapsing fever of unknown origin developed into an epidemic affecting around 400,000 German soldiers and 800 000 allied troops stationed in Northern France between 1915 and 1918 [11]. In 1916, the German bacteriologist Hans Töpfer identified a *Rickettsia* microorganism in body lice from a patient with a similar type of relapsing fever [12]. The infectious agent was called *R. quintana,* later the name was changed to *Bartonella quintana*. The disease got the name trench fever, as it emerged in soldiers living for months/years under very poor hygienic conditions in the trenches of the battlefield [11]. Besides, the shelters in the trenches, where soldiers lived and slept, were extremely crowded, making the propagation of body and head lice very likely.

Anecdotal reports mention that trench fever occurred in relatives of returning soldiers [11]. However, this does not indicate that *B. quintana* was only transferred by body lice: as a general rule, soldiers immediately changed infested clothes when they returned home, so that the likelihood of the presence of body lice was low. In contrast, head lice may have gone unnoticed for some time, because only about one-third of infested individuals develop symptoms.

Between 1940 and 1942, a devastating typhus epidemic occurred in the Warsaw Ghetto causing an estimated 16,000–22,000 deaths [13]. The Ghetto was declared as a “restricted infectious disease area” by the German military administration and was fenced off with a 3 m high wall. The residents became trapped in a kind of oversized prison from which escape was not possible. In the hermetically sealed area, 445,000 people lived temporarily, corresponding to 131,000 inhabitants per square kilometer—the highest population density ever recorded. The hygienic conditions under which the people lived were extremely poor: water pipes and sewers were destroyed by bombs and toilets did not work properly. Water was scarce, and the majority of the public bathhouses was closed down. As soon as a case of typhus became known, the whole family was forced into quarantine for two weeks, which in turn increased crowding and made adequate hygiene impossible.

According to the biography of Dr. Ludwig Hirszfeld, a physician and microbiologist who spent 3 years in the Ghetto and who developed a method to cultivate *R. prowazekii*, the infestation with head and body lice was omnipresent [14]. Supposedly, in the living conditions of the Ghetto, infection of body and head lice through sucking of blood from a patient with typhus had the same likelihood. Interestingly, the incidence rate of typhus started to decrease when hygienic measures directed against both body and head lice were implemented [13].

## Evidence from experimental studies

Since the pioneering work of Nicolle in 1912 cited in [15], an impressive number of experimental studies showed that body lice can transmit an array of highly pathogenic bacteria (Table 1). In contrast, rather few experimental studies were performed with head lice. In 1912, Goldberger and Anderson [16] collected head lice from the hair of patients with typhus and used these lice to infect rhesus monkeys with *R. prowazekii*. Their findings were later confirmed by Murray and Torrey [17], who infected head lice with *R. prowazekii* by feeding these lice on a rabbit infected with this pathogen. Using labelled antibodies, the authors demonstrated that 6 days after feeding head lice passed infective Rickettsiae in their faeces. These findings were confirmed by Robinson et al. 2003 [18]. Recently, using laboratory-raised head and body lice, it was shown that after oral feeding with blood containing a defined number of *B. quintana* bacteria, the number of *B. quintana* present in the faeces was almost the same in body and in head lice [19]. However, the authors observed that the average viability of *B. quintana* became lower in head than in body lice during an observation period of 11 days. Whether this might have an impact on the transmission of *B. quintana* through contact with flea faeces was not investigated. These findings corroborate previous observation showing the presence of *B. quintana* DNA in head lice [20–22].

## Evidence from epidemiological studies

Since 2015, an impressive number of epidemiological studies showed that head lice are infected with a panel of bacterial pathogens. In one group of studies, participants were infested only with head lice and lived in different settings in Thailand, Algeria, Mali, Democratic Republic of Congo, and Madagascar (Table 2). Study sites were selected, such that *Borrelia recurrentis*, *Bartonella quintana* or *Coxiella burneti* were expected to occur in the local population. *B. quintana* was detected in head lice from inhabitants of rural areas in Madagascar and Mali [23, 24], *C. burneti* in head lice collected from school children in Algiers as well as in refugees and healthy individuals from Niger [24, 25]. *B. recurrentis* was detected in head lice collected from pygmies living in the tropical rainforest in the Democratic Republic of Congo [26] and in inpatients with louse-borne relapsing fever in Ethiopia [27]. In addition, 14 different species of Acinetobacter were identified in head lice, including hitherto unknown species [23, 25, 26, 28–30].

In the second group of studies, participants were infested with both head lice and body lice (Table 3). Here, settings were selected, such that *B. recurrentis*, *B. quintana*, *R. prowazekii* or *Y. pestis* were likely to circulate in the local population. *B. quintana*-DNA was detected in head lice collected from healthy individuals in the Democratic Republic of Congo [4, 31], Ethiopia [32], Nepal [21] and other African countries [33]. In a study on trench fever in Ethiopia, either head lice or body lice were infected with *B. quintana* [32]. Bonilla et al. [22] examined 138 homeless adults from San Francisco for the presence of body lice and head lice. 23.9% of the individuals were infested with body lice, 8.7% with head lice, and 4.3% had co-infestations. *Bartonella* DNA was detected in 33.3% of body lice-infested persons and in 25.0% of head lice-infested persons. When pools of lice were compared, the positive rate for *B. quintana* DNA was 5.0% in body lice and 8.3% in head lice.

In a study on *B. quintana* in head lice of Nepalese children Sasaki et al. [21] identified *B. quintana* in 0% of head lice recovered from healthy school children living in a rural town, but in 12.5% of head lice collected from the scalp of street children. Homeless children were significantly more often co-infested with body lice than children from the rural town.

40.3% of head lice from double-infested patients with louse-borne relapsing fever in Ethiopia carried *B. recurrentis* [27]. *B. recurrentis* was also identified in head lice of pygmies from the Democratic Republic of Congo [28].

*Yersinia pestis* was detected in head lice from double-infested individuals living in a *Y. pestis* endemic area [4, 31]. In the latter study 2.9% of the head lice were infected with *Y. pestis* in contrast to 1.3% of body lice.

*Acinetobacter spp*. were recovered from head lice collected from school children in France [3, 29] and healthy individuals living in Southeast Ethiopia [34]. In the double-infested Ethiopians 47% of the head lice and 71% of the body lice carried *A. baumannii* [34]. None of the studies was designed to determine whether the infection rates in head lice and body lice were significantly different.

## Evidence from louse-borne relapsing fever imported to Europe by refugees

When in 2015 thousands of asylum-seeking young Africans reached Europe, louse-borne relapsing fever emerged in intensive care units in Italy, Switzerland, The Netherlands, Germany and even Finland [35–42]. Diagnosis of louse-borne relapsing fever was confirmed by PCR in all cases. Twenty-three of the patients were from Somalia and three from Eritrea. They had all taken a similar travel route through Kenya, Ethiopia, the Sudan, Libya and Italy. Before crossing the Mediterranean Sea, they had stayed in Libya in a concentration-camp-like setting for up to a year. Head lice as well as body lice are likely to occur in such settings and transmission of *B. recurrentis* must have been common [43].

Louse-borne relapsing fever developed between 3 days and 3 years after the refugee had reached the city, where he fell sick and was admitted to the hospital [40, 44]. Only in one case a louse was detected. It was judged to be a body louse by macroscopic examination [41]. As head lice were not systematically looked for, the presence of head lice cannot be ruled out. The observation that refugees spent a few weeks (in some cases up to 1 year) in Rome or Turin in crowding conditions before developing relapsing fever, and the fact that the clothes of refugees arriving in Italy had to be completely changed immediately after arrival, make it likely that head lice were responsible for the transmission of *B. recurrentis* [36, 44].

## Conclusions and discussion

Head lice occur worldwide in children and their caretakers with prevalences up to 40% in resource-poor settings [45]. In contrast, body lice only occur in homeless people and refugees, or occur in impoverished communities situated at high altitude in rural Ethiopia or in prisons in low-income countries, where hygienic conditions are poor, and crowding is common [46–48]. In 1999, Raoult and Roux assumed that body lice most probably can “transmit any agent ingested with a blood meal and capable of surviving in the insect’s midgut” [49]. Actually, there is plenty of evidence from experimental studies that body lice can transmit a broad array of pathogens (Table 1). Hitherto, only for *R. prowazekii* it was demonstrated that head lice can actively transmit this pathogen [15–17]. However, these studies were done in a time when no animal model suitable for head lice existed.

It should be noted that transmission of *B. quintana* is particular in the sense that faecal material of lice containing the pathogen has to be actively introduced into the skin, usually by scratching. Intense itch leading to scratching is typical for both types of lice infestation. Particularly in children with head lice infestation, excoriations of the scalp caused by scratching are common (H. Feldmeier, unpublished observation 2010). The odds that *B. quintana* is transmitted through scratching might, therefore, be higher.

Data from epidemiological studies indicate that head lice carry the same panel of bacterial pathogens as body lice, namely, *R. prowazekii*, *B. quintana*, *B. recurrentis*, *Y. pestis*, *C. burneti* and even *Acinetobacter spp*. (Tables 2, 3). Although in some studies the proportion of body lice infected with a defined pathogen seemed to be higher than in head lice, in other studies the contrary was the case [3, 4, 20, 21, 27, 31–33, 50]. Since none of the studies was powered to demonstrate a higher risk for infection of body lice compared to head lice with a defined pathogen, no conclusion can be drawn by comparing proportions of infected body lice versus infected head lice as done by the authors. Besides, the proportion of head lice infestation to body lice infestation in co-infested individuals is not a constant and depends on the setting in which the study was performed as well as on cultural attitudes, such as length and thickness of hair or type of clothing. This is highlighted by studies from the Democratic Republic of Congo [4, 31], Ethiopia [32], Nepal [21], and homeless adults from San Francisco [22].

Moreover, some of the epidemiological studies seem to have been biased, because the differentiation of body and head lice was done based on morphological criteria or at which part of the body the louse was identified (body versus scalp). Studies performed on head and body lice from nine different African countries showed surprising morphological difference even within a country [33, 34]. Not only the size and the body proportion differed from country to country, but also the color. Whereas all head lice from Senegal, Madagascar and Ethiopia were of black color, body lice were black in Madagascar and Rwanda, brown in Kenya and grey in Ethiopia. Body and head lice from Ethiopia and Rwanda were black and indistinguishable by body size [33]. Assumably, in older studies head lice were mistaken for body lice, making a conclusion of their susceptibility to infection with a defined pathogen impossible [20].

Historical observations indicate that body lice and head lice could have transferred *Y. pestis*, *B. quintana*, and *R. prowazekii* from sick to healthy individuals [6–9, 11, 13, 14, 46]. In 1998, a puzzling case was reported concerning a traveler from Algeria who developed typhus after returning to France [51]. No body lice were found, but the patient did recall having had intensive pruritus of the scalp during his stay in Algeria indicating the presence of head lice.

It goes without saying that the presence of a bacterial pathogen in an arthropod is not sufficient to conclude that it can transmit to humans. Whether body lice and head lice actually have the same potential to transmit bacterial pathogens cannot be ascertained from the existing data. Further research is needed using standardized experimental models.

## Data Availability

Data sharing is not applicable to this article as no data sets were generated or analyzed during the current study.
